# Complementation of a mutation in *CpSRP43* causing partial truncation of light-harvesting chlorophyll antenna in *Chlorella vulgaris*

**DOI:** 10.1038/s41598-017-18221-0

**Published:** 2017-12-20

**Authors:** Won-Sub Shin, Bongsoo Lee, Nam Kyu Kang, Young-Uk Kim, Won-Joong Jeong, Jong-Hee Kwon, Byeong-ryool Jeong, Yong Keun Chang

**Affiliations:** 1 Advanced Biomass R&D Center (ABC), Korea Advanced Institute of Science and Technology (KAIST), 291 Daehak-ro, Yuseong-gu, Daejeon 34141 Republic of Korea; 2 Department of Chemical and Biomolecular Engineering, Korea Advanced Institute of Science and Technology (KAIST), 291 Daehak-ro, Yuseong-gu, Daejeon 34141 Republic of Korea; 3 Plant Systems Engineering Research Center, Korea Research Institute of Bioscience and Biotechnology (KRIBB), 125 Gwahak-ro, Yuseong-gu, Daejeon 34141 Republic of Korea; 4 Department of Food Science and Technology, Institute of Agriculture and Life Science, Gyeongsang National University, Jinju, 660–701 Republic of Korea

## Abstract

Photosynthesis of microalgae enables conversion of light energy into chemical energy to produce biomass and biomaterials. However, the efficiency of this process must be enhanced, and truncation of light-harvesting complex (LHC) has been suggested to improve photosynthetic efficiency. We reported an EMS-induced mutant (E5) showing partially reduced LHC in *Chlorella vulgaris*. We determined the mutation by sequencing the whole genome of WT and E5. Augustus gene prediction was used for determining CDS, and non-synonymous changes in E5 were screened. Among these, we found a point mutation (T to A) in a gene homologous to chloroplast signal recognition particle 43 kDa (CpSRP43). The point mutation changed the 102nd valine to glutamic acid (V102E) located in the first chromodomain. Phylogenetic analyses of CpSRP43 revealed that this amino acid was valine or isoleucine in microalgae and plants, suggesting important functions. Transformation of E5 with WT CpSRP43 showed varying degrees of complementation, which was demonstrated by partial recovery of the LHCII proteins to the WT level, and partially restored photosynthetic pigments, photosynthetic ETR, NPQ, and growth, indicating that the V102E mutation was responsible for the reduced LHC in E5.

## Introduction

Microalgae are photosynthetic eukaryotes (excluding plants) known for their high photosynthetic efficiency and potential for use in the production of biomass and biomaterials. However, they are far less efficient relative to their theoretical photosynthetic potential partly due to an oversized photosynthetic antenna, which functions as a light-harvesting complex (LHC)^1^. Microalgae have evolved to cope with low light levels and competition with other organisms, which have led to an increase in the LHC size. This results in shading effects in an artificial high-density culture, contributing to the reduced photosynthetic efficiency^2^. LHCs are composed of various photosynthetic pigments and pigment-binding proteins. In higher plants and green algae, LHCI associated with PSI and LHCII associated with PSII consist of LHCA and LHCB proteins, respectively^3^.

The proteins needed for the assembly of LHCs and photosystems are guided and integrated into the developing thylakoid membrane by the chloroplast signal recognition particle (CpSRP) pathway^4,5^, which is similar to the signal recognition particle (SRP) pathway in bacteria^6^. Nuclear-encoded LHC proteins are targeted to chloroplasts via their transit peptide and are imported into the chloroplast stroma. CpSRP43 and CpSRP54 form a transit complex together with imported LHC proteins^4,7^, and is recognized by the signal recognition receptor CpFTSY. This LHC-CpSRP43-CpSRP54-CpFTSY complex is guided to the chloroplast SRP insertase ALB3 protein^8,9^, and the LHC protein is integrated into the thylakoid membrane. The CpSRP complex is disassembled for another cycle of LHC protein integration. Chloroplast-encoded proteins such as PSII reaction-center proteins are integrated into the thylakoid membrane via the co-translational pathway, which involves CpSRP54, ALB3, CpSECY and potentially CpFTSY^4,10,11^.

Recent studies of green algae show that the functions of the proteins involved in the CpSRP pathway differ from those of higher plants according to analyses of knockout mutants of CpSRP pathway-related genes in *Chlamydomonas reinhardtii* ^4,9,12–14^. These knockout mutants have phenotypes of truncated light-harvesting chlorophyll antenna (TLA) mutations, which show reduced levels of chlorophylls and LHC proteins. These TLA mutations have been suggested as a strategy for the genetic engineering of microalgae to improve photosynthetic efficiency^1,15^. Microalgae have evolved to possess extensive light-harvesting chlorophyll antenna to maximize light absorption in light-limiting environments^16^. This process is advantageous for the survival of cells in an environment in which they must compete with other organisms. However, in high-density monocultures for the production of biomass and other target products, the maximized light absorption by oversized LHCs leads to the wasteful dissipation of light energy into heat via non-photochemical quenching (NPQ) and uneven light distributions in cell cultures^17,18^. TLA mutants showed improved photosynthetic activity and accelerated growth under high light conditions by alleviating the over-absorption of light and by allowing greater light penetration^14,15,19,20^. Moreover, recent studies have demonstrated that the application of a TLA strategy can enhance crop yields^21,22^.

We previously reported a TLA mutant (E5) generated by ethyl methanesulfonate (EMS) mutagenesis in *Chlorella vulgaris* which showed improved biomass productivity under high light conditions^20^. Identification of the responsible gene in E5 can provide a valuable genetic resource for potential target genes to improve biomass production in *Chlorella* and other microalgae. By sequencing the whole genome of WT and E5, and with Augustus gene modeling, we were able to identify non-synonymous mutations in E5. Among these, a point mutation was found in CpSRP43, and complementation analyses by transforming E5 with the WT gene confirmed that this mutation was responsible for the TLA phenotype in E5. To the best of our knowledge, the determination and complementation of the random mutation is the first in *Chlorella*, except for the complementation of the nitrate reductase marker^23,24^.

## Results

### Identification of a mutation in the truncated light-harvesting antenna mutant of *C*. *vulgaris*

We generated a TLA mutant (E5) via EMS mutagenesis in *C*. *vulgaris* UTEX265 and reported its phenotypes, including reduced photosynthetic pigments and LHC components^20^. We sequenced the whole genome of WT and ET to determine the responsible mutation(s). Coding sequences were predicted with Augustus gene prediction on the reference genome of *C*. *vulgaris* UTEX395 (GenBank assembly accession: GCA_001021125.1). E5 showed 63 single nucleotide polymorphisms (SNPs) compared to WT, and among these, 20 were in the coding sequence (CDS) (Table S2). Because EMS mainly induces base pair changes^25,26^, single-nucleotide polymorphisms (SNPs) were analyzed in E5. Compared to the *C*. *vulgaris* wild type, 63 SNPs were detected in the E5 genome, and among them, 20 SNPs were in the coding sequence (Table S2). The 20 SNPs were then determined as non-synonymous or synonymous SNPs with respect to the CDS of the *C*. *vulgaris* UTEX395 reference genome and were annotated (Table S3). When comparing the *C*. *vulgaris* wild type and E5, synonymous SNPs were initially excluded from the mutation candidates. Among the remaining SNPs, a non-synonymous SNP locus showed high homology with a gene encoding the *chloroplast SRP43* (*CpSRP43*) gene in *Chlamydomonas reinhardtii*, which is known to be responsible for the transport of light-harvesting proteins (LHCPs) into the thylakoid membrane^13,27^. A nucleotide T in the wild type was changed to an A in the E5 mutant, which led to an amino acid change from the 102nd amino acid valine (V) to glutamic acid (E: V102E). Several studies have reported that the knockout of *CpSRP43* gene resulted in reduced amounts of chlorophyll and TLA phenotype in green algae and higher plants^13,28,29^, similar to the E5 mutant^20^. Because the mutation in E5 did not result in the knockout of the *CpSRP43* gene in *C*. *vulgaris* or generated a premature CpSRP43 protein by creating a stop codon, we hypothesized that the single amino acid change may induce loss-of-function phenotypic changes in E5.

### Analysis of the CpSRP43 protein sequence of *C*. *vulgaris*

**Figure 1:**
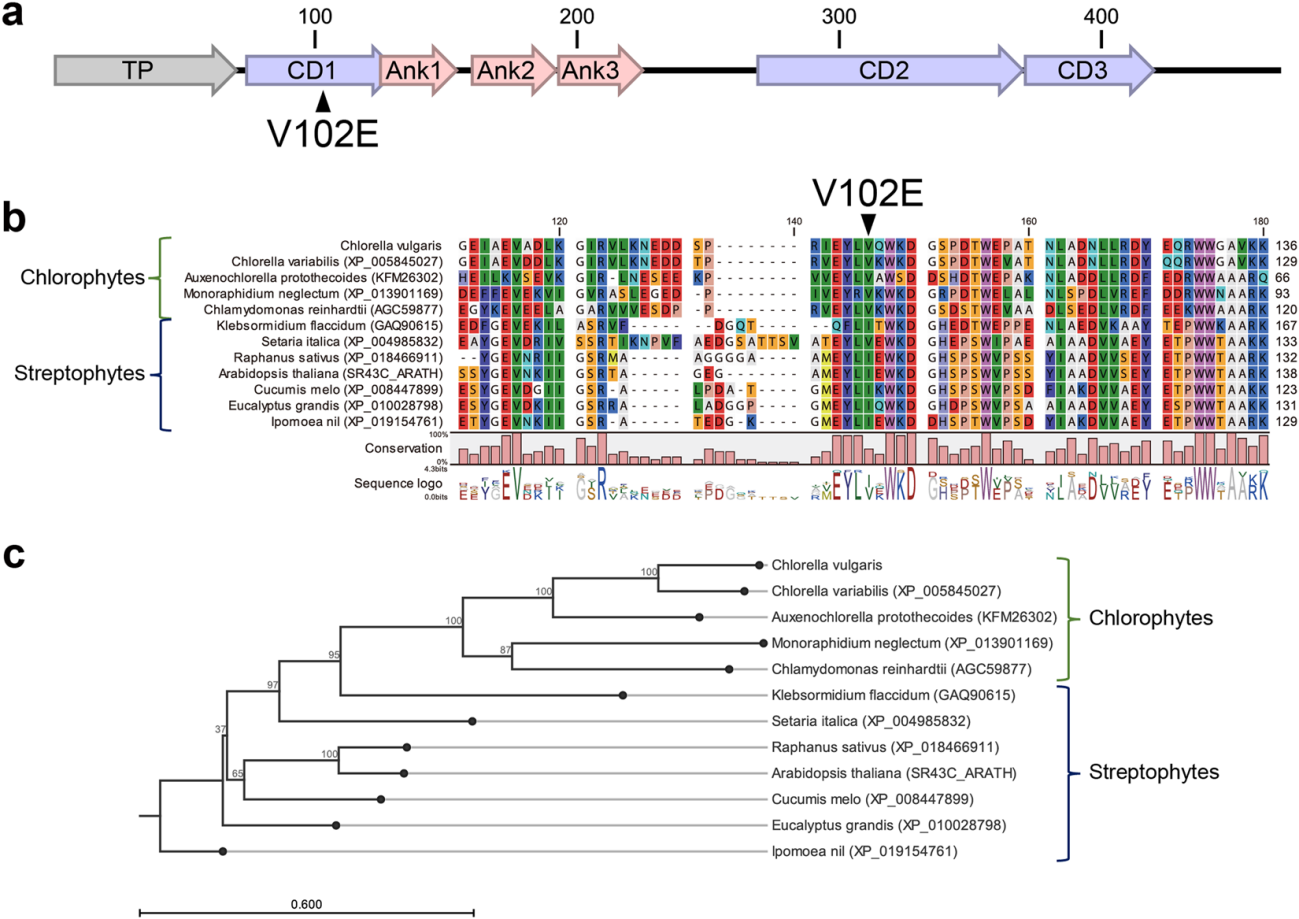
Sequence analysis of CpSRP43-homologous protein in *C*. *vulgaris*. (**a**) Conserved domains in the CpSRP43-homologous protein of *C*. *vulgaris*. A mutation in E5 changed the 102^nd^ valine (V) to glutamic acid (E). (**b**) Alignments and (**c**) maximum likelihood phylogenetic tree of CpSRP43-homologous proteins from various organisms.

We cloned CDS of *CvCpSRP43* from the cDNA of *C*. *vulgaris* UTEX265, which was sequenced to confirm the correct amplification. The genomic sequence was 3643 bp containing nine exons, and the CDS encoded 468 amino acids. The first 70 amino acids were predicted to be a transit peptide (TP) required for targeting to the chloroplast, based on ChloroP^30^ (http://www.cbs.dtu.dk/services/ChloroP/) and TargetP^31^ (http://www.cbs.dtu.dk/services/TargetP/) (Fig. 1a). The CpSRP43 protein of *C*. *vulgaris* contained three chromodomains (CD1, amino acids 74–131; CD2, amino acids 269–370; CD3 amino acids 371–420) and three ankyrin repeats (ANK1, amino acids 125–154; ANK2, amino acids 160–192; ANK3, amino acids 193–225), as predicted by InterPro (https://www.ebi.ac.uk/interpro/) (Fig. 1a). A single nucleotide change in E5 (GTG in WT, while GAG in E5) resulted in a change of the 102^nd^ amino acid from valine (V) to glutamic acid (E), which was located in CD1.

In order to verify the importance of V102E, we aligned CpSRP43 protein sequences from plants and microalgae, and found that the valine residue was located in the conserved region of CD1 (Fig. 1b and S1). Chlorophytes mainly possessed valine in this position, while streptophytes possessed valine or isoleucine, as represented by two bulky aliphatic amino acids. Based on the alignment data of the whole CpSRP43 sequences (Fig. S1) and the phylogenetic tree (Fig. 1c), the CpSRP43 of *C*. *vulgaris* was closest to that of *Chlorella variabilis*. Overall, CpSRP43s were grouped according to the lineages of the chlorophytes and streptophytes (Fig. 1c).

### Complementation of E5 with WT *CvCpSRP43*

The CDS of *CpSRP43* was amplified from *C*. *vulgaris* UTEX265 and cloned into the 29B HSP70P-CFP-NosT vector with the pUC19 backbone to replace the *CFP* gene, and a hygromycin-resistant gene cassette was inserted next to it Fig. S2a). A linearized vector was introduced to the E5 mutant by electroporation, and the hygromycin-resistant colonies were selected. Introduction of the vector into the cell was confirmed by PCR, amplifying 938 bp spanning the 29B HSP70 promoter and the CpSRP43-flag region in the vector. The target bands were only detected in four complemented strains (C2, C3, C4 and C6), while the 18 S rDNA bands were detected in all samples (Fig. S2a). These four complemented strains (C2, C3, C4 and C6) were subjected to further analysis.

### Characterization of photosynthetic pigments in the complemented strains

**Figure 2:**
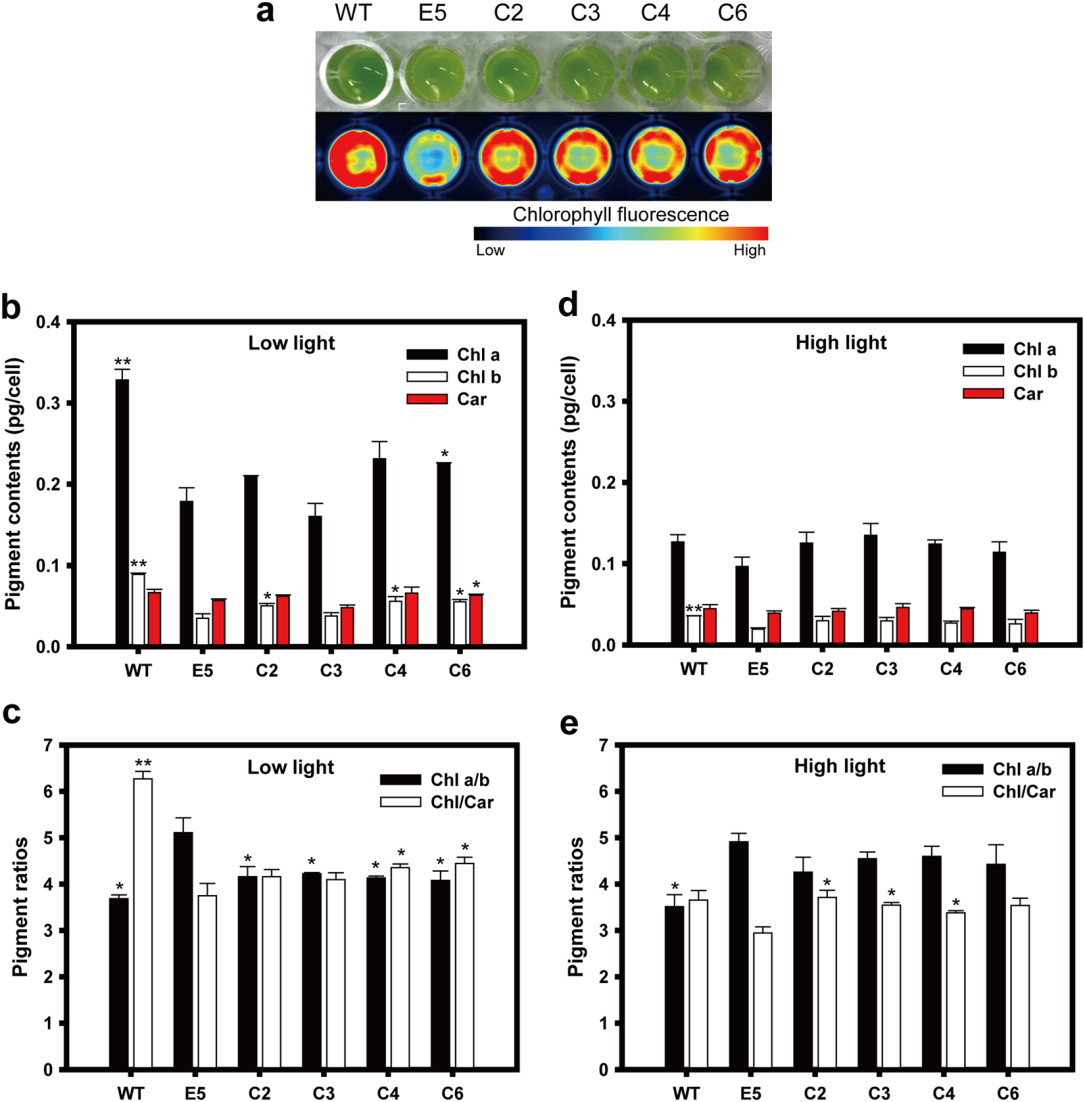
Analysis of chlorophyll fluorescence and pigments. (**a**) Visible phenotypic differences and chlorophyll fluorescence as visualized by blue epi-illumination and a 695/55 nm filter using a ChemiDoc system (Bio-Rad, USA). (**b**) Pigment contents and (**c**) pigment ratios of *C*. *vulgaris* wild type (WT), E5 and complemented strains grown under a low light condition (50 μmol photons m^−2^ s^−1^). (**d**) Pigment contents and (**e**) ratios of cells grown under a high light condition (300 μmol photons m^−2^ s^−1^). Total chlorophyll content (sum of Chl *a* and Chl *b*) was used to calculate Chl/Car ratio. The data represent the average of samples and the error bars indicate the standard error (*n* = 2). Significant differences between E5 and other strains (WT and complemented strains) were determined by Student’s *t* tests and are indicated by asterisks (*P < 0.05, **P < 0.01, ***P < 0.001).

The photoautotrophically grown *C*. *vulgaris* WT, E5, and the complemented strains (C2, C3, C4 and C6) were harvested in the mid-exponential growth phase and concentrated to have identical cell density levels (2 × 10^8^ cells/mL). Visual inspection of cells revealed that the complemented strains recovered their dark green color, which was comparable to that of WT and darker than that of E5 (Fig. 2a). Identical cell suspensions were used to measure the chlorophyll fluorescence intensity using ChemiDoc (Bio-Rad, USA), and the complemented strains showed an intermediate level between WT and E5 (Fig. 2a). The contents of chlorophyll *a* and *b* per cell were higher in C2, C4 and C6 than in E5 (Fig. 2b), and their carotenoid (Car) contents were also recovered. The ratio of chlorophyll *a* to *b* (Chl *a*/b) was significantly lower in all complemented strains compared to that of E5, while their Chl/Car ratios were higher than that of the E5 but lower than WT (Fig. 2c). As an exception, the C3 strain did not show increased levels of chlorophylls, but its Chl *a*/*b* ratio was significantly low compared to E5, similar to other complemented strains. Contents of photosynthetic pigments were reduced under the high light conditions compared under the low light conditions, particularly chlorophyll contents were reduced to about 50% level (Fig. 2d) and the Chl/Car ratios were also reduced (Fig. 2e). Such reduction of photosynthetic pigments under high light has been reported as a photoacclimation in other photosynthetic organisms^32^. In general, the Chl *a*/*b* ratio of the TLA mutants increases because chlorophyll *b* mainly binds to the peripheral light-harvesting antenna in green algae and higher plants^19,33–35^. Therefore, the decreased Chl *a*/*b* ratios of the complemented strains suggest that their peripheral LHCPs were partially recovered.

### Molecular analysis of the complemented strains

**Figure 3:**
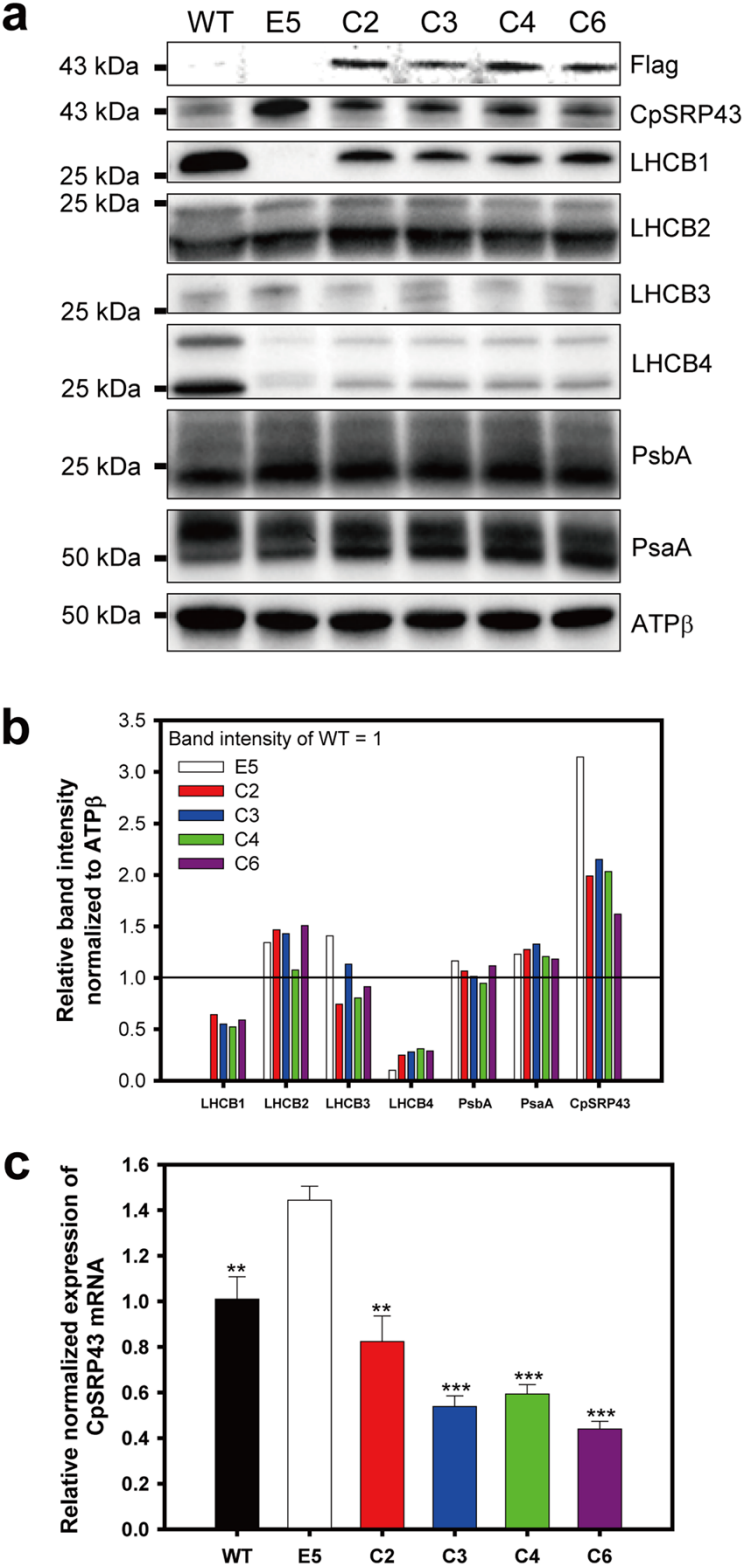
Molecular analysis of *C*. *vulgaris* wild type, E5 and complemented strains. (**a**) Western blot analysis with various antibodies for light-harvesting complex proteins. Chemiluminescent signal was detected with ChemiDoc system (Bio-Rad, USA) and expected bands were cropped. Full-length blots and gel images were presented in Fig. S3. (**b**) Relative band intensities normalized to ATPβ. (**c**) Expression levels of the total CpSRP43 mRNA as measured by qRT-PCR. The data represent the average of samples and the error bars indicate the standard error (*n* = 3). Significant differences between E5 and other strains (WT and complemented strains) were determined by Student’s *t* tests and indicated by asterisks (*P < 0.05, **P < 0.01, ***P < 0.001).

To understand the molecular characteristics of the complemented strains, western blots and qRT-PCR were conducted for WT, E5 and the complemented strains (Fig. 3 and S3). Western blots were performed to analyze the LHCPs and reaction center proteins in the complemented strains. We used antibodies raised against *Arabidopsis* LHCB1, LHCB2 and LHCB3, and were able to detect proteins with expected sizes and distinctive expression patterns (Fig. 3a). However, it should be noted that these homologs have not been identified in *C*. *vulgaris* due to incomplete genome information. Other conserved proteins, including CpSRP43, LHCB4, PsaA and PsbA, were also detectable using antibodies raised against *Chlamydomonas* proteins. The β subunit of ATP synthase was used as a loading control and was used to normalize the quantities of other proteins. PsbA and PsaA (the reaction center proteins of PSII and PSI, respectively) were relatively even in all strains and did not show any differences compared to WT (Figs 3a,b and S3). LHCB1-like and LHCB4-like proteins were expressed at very low levels in E5, as reported in Shin *et al*.^20^. Expressions of these homologs were increased in complemented strains but lower than that of WT, indicating partial complementation (Fig. 3a and b). This partial complementation pattern of antennal proteins was also observable directly from gel images near 25 kDa due to their abundant expression (Fig. S3j). C6 showed most expression of antennal proteins while C2 showed the least, even though this banding pattern did not match the western blots that showed even distribution of antennal proteins in complemented strains. The discrepancy suggests that there were more proteins missing in complemented strains. Additional LHC-like proteins, the LHCB2- and LHCB3-like proteins, were not affected in either E5 or the complemented strains. Overall, complemented strains showed partial recovery of only defective LHCPs in E5, while the remaining antennal and photosynthetic proteins were not affected. It should be noted that detection of *Chlorella* proteins using heterologous antibodies resulted in extra bands in addition to expected ones (Fig. S3), which leaves room for detection of unrelated proteins until confirmation by reproducible reports.

We also analyzed the expression of the CvCpSRP43 protein and RNA (Fig. 3). We used a polyclonal antibody against *Chlamydomonas* CpSRP43, as reported in Shin *et al*.^36^, for western blotting. Intriguingly, E5 showed an increased expression level of CvCpSRP43 compared to that by WT (Fig. 3b), possibly due to positive feedback regulation, which compensated for the lack of a *CvCpSRP43* function in E5. Complemented strains showed reduced expression levels of CvCpSRP43, with these levels between those of WT and E3, consistent with the partial complementation results shown for antennal proteins. RNA expression, revealed via qRT-PCR, also showed a pattern similar to those of proteins, with E5 highest (Fig. 3c). However, complemented strains were lowest in all cases, though the discrepancy with the protein expression is not clear.

### Light-dependent photosynthetic electron transport rate and non-photochemical quenching

**Figure 4:**
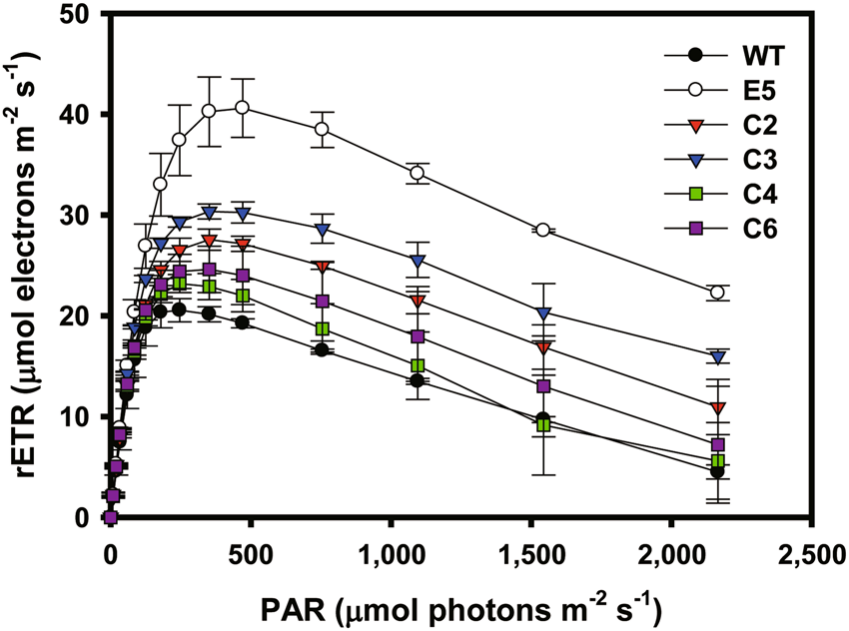
Effect of complementation on photosynthesis. Light-dependent curves of the relative electron transport rate (rETR). The step width of each data point was 2 min. The data represent the average of samples and the error bars indicate the standard error (*n* = 2).

To examine the effects of LHCB1- and LHCB4-like proteins recovery on photosynthesis, the relative electron transport rate (rETR) and quantum yield of non-photochemical quenching [Y(NPQ)] of each strain were analyzed based on *in vivo* chlorophyll fluorescence analyses. The rETR and Y(NPQ) values were recorded while increasing the light intensity, and the level of significance was determined by the Student’s *t* test, where p < 0.05 indicated significance between E5 and the complemented strains under high light intensity. We previously observed higher rETR and lower Y(NPQ) values in E5 compared to those of WT with a high intensity level of photosynthetically active radiation (PAR)^20^. All four complemented strains showed a consistent trend of decreased rETR and increased Y(NPQ) to an intermediate level between WT and E5 (Fig. 4 and S4). All strains showed similar rETR outcomes at low light intensities (below 100 μmol photons m^−2^ s^−1^), but E5 showed much higher rETR values at high light intensities (over 200 μmol photons m^−2^ s^−1^), while the outcomes for the complemented strains were between those of E5 and WT. This pattern was maintained at a higher light intensity up to 2,000 μmol photons m^−2^ s^−1^. The Y(NPQ) data also showed a trend toward partial complementation, where the average values were between those of WT and E5 under high light intensity levels (100–800 μmol photons m^−2^ s^−1^) (Fig. S4). Differences among the Y(NPQ) values at the dark recovery stage were not significant, suggesting that the photoprotection mechanisms operated normally to dissipate energy.

### Photoautotrophic growth curves at different light irradiances

**Figure 5:**
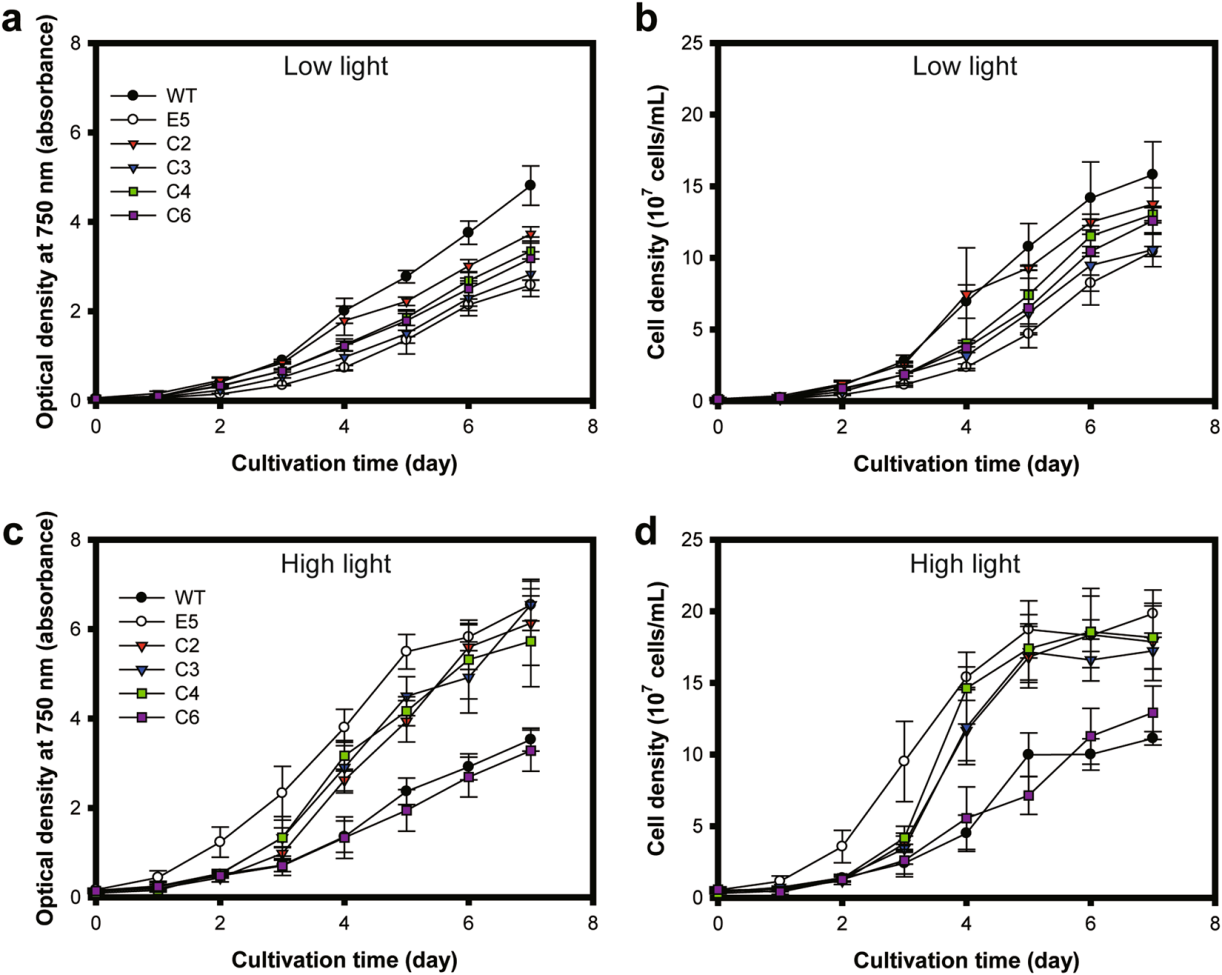
Cellular growth curves under different light intensities. Cellular growth of *C*. *vulgaris* wild type (WT), E5 and complemented strains (C2, C3, C4 and C6) under (**a**,**b**) a low light condition (50 μmol photons m^−2^ s^−1^) and (**c**,**d**) a high light condition (300 μmol photons m^−2^ s^−1^), as monitored with the optical density at 750 nm (*OD*_750_) and the cell density. The data represent the average of samples and the error bars indicate the standard error (*n* = 2 for the low light conditions, *n* = 3 for the high light conditions).

We examined whether the complementation of E5 ultimately affected cellular growth under different light conditions. Cells were grown photoautotrophically in column photobioreactors under continuous illumination with low light (50 μmol photons m^−2^ s^−1^) or high light (300 μmol photons m^−2^ s^−1^), as shown in Fig. 5. Cellular growth was monitored by measuring the optical density at 750 nm and the cell density. Previously, E5 achieved higher biomass productivity in flat-panel photobioreactors under a high light condition, while the productivity of E5 was lower than that of WT under low light conditions^20^. This trend was maintained in this study, in which a column photobioreactor with a working volume of 200 mL was used. With the low light condition, the growth of E5 was lower than that of WT, and all complemented strains were between E5 and WT when the optical densities were measured (Fig. 5a). Growth analyses based on the cell density were obscured due to high error bars, but they showed patterns similar to those of the optical density (Fig. 5b). In contrast, under the high light condition, the growth of E5 was significantly better than that of WT, and the complemented strains showed growth outcomes between WT and E5 (Fig. 5c). In particular, C6 was close to WT, and C3 was closest to E5, revealing the lowest level of complementation, consistent with other complementation phenotypes. Growth analyses based on cell density were consistent with those of the optical density, albeit with higher error bars most likely due to the intrinsic error-prone estimation of the cell density (Fig. 5d). Overall, partial complementation was consistently observed in the growth results as well as in other phenotypes, including the recovery of photosynthetic pigments (Fig. 2), the LHCPs (Fig. 3), and the photosynthetic parameters (Fig. 4).

## Discussion

Photosynthetic organisms usually have large arrays of LHC and photoprotection mechanisms to survive under fluctuating light conditions^16,17,37^. However, these mechanisms can be a main bottleneck and thus reduce photosynthetic efficiency under artificial cultivation conditions. Truncation of LHC has been suggested as a solution for these problems, and previous studies have shown improvements of photosynthetic efficiency and biomass productivity by alleviating the over-absorption of light on the culture surface and achieving a better light distribution over the whole culture in the model microalga *Chlamydomonas reinhardtii* ^1,4,18,19,38,39^. We demonstrated that the antenna truncation strategy also worked in a commercial microalgal species, *C*. *vulgaris* ^20^. This study determined a SNP at the *CvCpSPR43* locus which was responsible for the TLA mutant phenotype in E5. Nucleotide transversion from T to A was induced by an EMS treatment to generate the E5 mutant^25,26^, resulting in an amino acid change from V to E (V102E) located in CD1 of CvCpSRP43.

The reduction of the LHCPs in E5 was limited to LHCB1- and LHCB4-like proteins, which are major and minor peripheral LHCPs in PSII, respectively. It is interesting that the partial reduction of LHCPs in E5 is different from the CpSRP43 knockout mutant of *C*. *reinhardtii*, which shows severe reductions of all major and minor LHCPs in PSII, including LHCB1/LHCB2, LHCB3, LHCB4 and LHCB5^13^. The discrepancy may be caused by the nature of the mutations, as the point mutation in E5 is located in V102E in CD1, while that of *C*. *reinhardtii* is disrupted by insertional mutagenesis leading to complete knockout of the gene functions^13^. The valine residue together with the isoleucine found mostly in plants is conserved and may be critical for certain functions. It is tempting to speculate that this amino acid or CD1 is involved in interactions with certain LHCPs. Thus far, it is known that the deletion of the CD1 domain from CpSRP43 increases the GTP hydrolysis activity^40^, suggesting that CD1 negatively regulates GTP hydrolysis by the CpSRP43/CpSRP54/CpFtsY complex^41–45^. Guanine nucleotide binding by CpSRP43/CpSRP43/CpFtsY releases LHCP from the CpSRP complex, and GTP hydrolysis is required for the recycling of CpSRP from CpFtsY^46^. Given these findings, it is conceivable that the V102E mutation in E5 somehow reduced the GTPase activity and disturbed the complete assembly of the light-harvesting antenna. It has also been reported that CD1 is rigidly fused to the neighboring ankyrin repeats that bind LHCPs^47^. It is therefore possible that the negative charge in V102E might have negative effects on interaction with the LHCB1- and LHCB4-like proteins, though this possibility requires further studies.

It is also interesting to note that the amounts of the CvCpRRP43 protein and RNA were increased in E5, whereas they were decreased (or partially recovered) in the complemented strains (Fig. 3). This is reminiscent of a type of feedback regulatory mechanism known as positive feedback loop regulation, where defective downstream processes can activate the expressions of the upstream cognate genes to compensate for defective functions. Such an example can be found in the increased expression of a forkhead transcription factor (FOXO3a) in patients diagnosed with Huntington’s disease^48^. This type of auto-regulatory mechanisms has not been reported in relation to microalgae, and is worthwhile to study this in more detail.

In complementation tests, recovery of the phenotypes occurred consistently at the intermediate level between WT and E5. Transformation of E5 with the WT *CpSRP43* gene would generate complemented strains harboring both mutated *Cpsrp43* and normal *CpSRP43*, which may interact negatively. In addition, the 29B HSP70 promoter was used to drive the expression of WT *CpSRP43* instead of the endogenous promoter in *C*. *vulgaris*. This transgenic expression of *CpSRP43* may not be sufficient to replace the mutated *Cpsrp43* completely in E5. However, all complemented strains showed consistent restoration of the TLA phenotypes (with small standard error), including photosynthetic pigments, LHCPs and photosynthetic parameters although small sample size (*n* = 2) was used to calculate P-value. This indicates that the V102E mutation in E5 was responsible for the TLA phenotypes of E5. This also suggests that the *CpSRP43* homologs are good targets for knock-down to improve biomass productivity in *Chlorella* ^20^. It should also be noted that there could be additional mutation(s) contributing to the phenotypes in E5, but this possibility cannot be pursued currently due to incomplete genomic information. SNPs in E5 should be reevaluated when genome sequencing is completed for *Chlorella vulgaris*.

Even though the TLA strategy has been demonstrated as a promising concept for improving microalgal productivity in lab-scale experiments under high light irradiances, potential caveats should be considered and studied for further application on outdoor large scales where the light irradiance is fluctuating. Under the low light conditions, reduced ability to absorb light in TLA mutants lowers photosynthetic productivity per cell. Considering light-limiting conditions, where photo-damage is not severe, photosynthetic growth would decrease as shown in this study (Fig. 5a and b). Besides, the TLA strategy is considered advantageous only under narrow range of conditions^49^. In this aspect, the TLA strategy might show better performance in continuous cultivation system because growth conditions can be easily controlled. For example, chemostat can be incorporated into the continuous cultivation system to maintain proper cell density resulting in maximum productivity, which can avoid light attenuation due to extremely dense culture. In fact, the potential for large scale application appears to be valid, since it has been reported that a TLA mutant can improve biomass productivity under the outdoor light condition^15^.

Conclusively, a single nucleotide change resulting in the V102E mutation in CD1 of CvCpSRP43was responsible for the TLA phenotype in E5. This was confirmed by the complementation experiments, where the complemented strains showed the partial recovery of the TLA phenotypes. In the identification and complementation experiments, we also made several interesting observations, including that of the LHCP-specific role for the 102^nd^ amino acid in CD1 and the possible positive feedback regulatory mechanism of the gene expression of *CvCpSRP43*, all of which deserve further studies. It should also be noted that the homologs of CvCpSRP43 would be good knock-down targets to improve biomass production in other industrial microalgae.

## Methods

### Cell culture conditions

*Chlorella vulgaris* UTEX265 was purchased from the algae culture collection at the University of Texas (Austin), and a random mutant, designated as E5, was previously generated by our group^20^. Cells were cultivated photoautotrophically in BG-11 medium^50^ at 25 °C with agitation at 120 rpm under continuous light (50 μmol photons m^−2^ s^−1^), and 3% (*v*/*v*) CO_2_ was supplied at 0.25 VVM (volume gas per volume medium per minute).

### DNA sequencing and gene prediction

Genomic DNAs of *Chlorella vulgaris* wild type and the E5 mutant were extracted according to a previously described method^51,52^. Briefly, cells were harvested in the mid-exponential growth phase, washed with a 50 mM ethylenediaminetetraacetic acid (EDTA) solution, and resuspended in 150 μL of deionized water (DW). Then, 300 μL of a SDS-EB solution (400 mM NaCl, 50 mM EDTA, 100 mM Tris-HCl, pH 8 and 2% sodium dodecyl sulfate (SDS)) was added and the sample was mixed. The genomic DNA was extracted using 500 μL of a mixture of phenol and chloroform (1:1, *v*/*v*). The supernatant was transferred to a new tube and the extraction step was repeated. The supernatant was washed with chloroform once, and the DNA was precipitated with two volumes of ethanol. The DNA pellet was washed with 70% ethanol and air-dried, after which it was resuspended in TE buffer (10 mM Tris-HCl, pH 8 and 0.2 mM DETA, pH 8). Whole-genome sequencing was conducted with an Illumina HiSeq. 2000 system (Illumina, USA) and the genomic DNA sequences were read with a paired-end sequencing platform (Seeders, Republic of Korea). For better quality, the SolexaQA package software (version 1.13) was used to eliminated poor-quality bases (Phred score < 20) with the Dynamic Trim module and to exclude short read lengths of < 25 bp with the LengthSort module. The trimmed reads were aligned to the *C*. *vulgaris* UTEX395 reference genome (GenBank assembly accession: GCA_001021125.1) using the BWA (0.6.1-r104) program^53^. The raw single-nucleotide polymorphisms (SNPs) and insertion/deletions (In/Dels) were detected using the SAMtools (0.1.16) program^54^, and consensus sequences were extracted. The SNP matrix was generated with SEEDERS in-house script^55^. For the gene prediction step, adapters were removed from the consensus sequence of *C*. *vulgaris* UTEX265 and quality trimming was conducted using Trimmomatic^56^, followed by *de novo* assembly with IDBA-UD (with *C*. *vulgaris* UTEX395 as a reference)^57^. Using the protein sequences of *Chlorella variabilis* NC64A as a reference parameter (GenBank assembly accession: GCA_000147415.1), genes were predicted by Augustus^58^. The predicted genes were subjected to tblastx (2.2.28+) (filter; E-value ≤ 1e-50, best hit) in the *C*. *vulgaris* UTEX395 reference genome. Synonymous and non-synonymous SNPs were then determined by searching for SNP loci included in the coding sequences.

### Complementation experiment

The *CpSRP43*-homologous gene in the *C*. *vulgaris* wild type was amplified using Phusion High-Fidelity DNA polymerase (NEB, USA) with the primers SRP43_Gibson_fwd and SRP43_Gibson_rev. The pUC19 vector was kindly provided by Dr. Won-Joong Jeong at the Korea Research Institute of Bioscience and Biotechnology (KRIBB), which contains the 29B HSP70 promoter-CFP gene-Nos terminator. The amplified fragments were assembled into the pUC19 vector amplified with the primers pUC19_fwd and pUC19_rev using a Gibson Assembly kit (NEB, USA) so that the gene expression was controlled by the 29B HSP70A promoter and Nos terminator. Another fragment of the b2TUB promoter-Aph7 gene-RbcS2 terminator, which confers resistance to hygromycin, was amplified with the primers Aph_Gibson_fwd and Aph_Gibson_rev and then assembled into the pUC19 vector backbone harboring the *CpSRP43* cassette, which was amplified with the primers Aph-pUC_back_fwd and Aph-pUC_back_rev. All fragments amplified by PCR were used after purification with a QIAquick gel extraction kit (Qiagen, Netherlands).

Cloned plasmids were delivered to the E5 mutant by electroporation. The E5 mutant was grown under continuous illumination (100 μmol photons m^−2^ s^−1^) and harvested in the mid-exponential growth phase. The harvested cells were resuspended in deionized water and moved to an electroporation cuvette with a 0.2 cm gap (Bio-Rad, USA). After icing for 10 min, electroporation was conducted using a Gene Pulser (Bio-Rad, USA), with cooling for a further 10 min. The cells were recovered for 24 hours in a TAP medium containing 40 mM of sucrose in the dark. After recovery, cells were spread on a TAP medium containing 1.5% (*w*/*v*) agar (Bacto^TM^ agar, BD Difco) supplemented with 200 μg/mL of hygromycin B. After four weeks, surviving colonies were selected and the insertion of the plasmids was confirmed by amplification of the target sequence (948 bp) with the primers S4_F and S4_R. The primers 18S_F1 and 18S_R1 were used to detect the housekeeping gene (the target sequence of 303 bp), 18 S rDNA, as a positive control. All primers used in this study are listed in Table S1.

### Photosynthetic pigment measurement

Photoautotrophically grown cells of the *C*. *vulgaris* wild type, E5 and the complemented strains were concentrated to 2 × 10^8^ cells/mL, and images were taken by a digital camera. After 15 min of dark adaptation, chlorophyll fluorescence images were taken by a ChemiDoc system (Bio-Rad, USA) with blue epi-illumination and a 695/55 nm filter.

The contents of chlorophyll *a* (Chl *a*), chlorophyll *b* (Chl *b*) and carotenoid (Car) were analyzed by a dimethyl sulfoxide (DMSO) extraction method^59^. Briefly, an amount of 1 mL of cells in the mid-exponential growth phase was centrifuged at 13,000 rpm for 2 min. After the cell pellet was suspended in 1 mL DMSO, the mixture was incubated at 60 °C for 40 min and then centrifuged again. The optical density of the supernatant was measured at 480, 649 and 665 nm (*OD*_480_, *OD*_649_, *OD*_665_) using a UV-Vis spectrophotometer (DU730; Beckman Coulter, Germany). The concentrations of the pigments were calculated by following equations:

$$\begin{array}{rcl}{\rm{C}}{\rm{h}}{\rm{l}}\,a\,[{\mu \mathrm{g\; mL}}^{-1}] & = & 12.19\times O{D}_{665}-3.45\times O{D}_{649}\\ {\rm{C}}{\rm{h}}{\rm{l}}\,b\,[{\mu \mathrm{g\; mL}}^{-1}] & = & 21.99\times O{D}_{649}-5.32\times O{D}_{665}\\ {\rm{C}}{\rm{a}}{\rm{r}}\,[{\mu \mathrm{g\; mL}}^{-1}] & = & [1000\times O{D}_{480}-2.14\times ({\rm{C}}{\rm{h}}{\rm{l}}\,a)\,-70.16\times ({\rm{C}}{\rm{h}}{\rm{l}}\,b)]/220\end{array}$$

The calculated concentrations were normalized with respect to the cell density of each sample, as determined by a Cellometer (Cellometer Auto X4, Nexcelom Bioscience, USA).

### Measurements of the expression levels of CpSRP43 mRNA and protein

Total RNA of each strain was extracted from cells harvested in the mid-exponential phase with RNeasy Plant mini kits (Qiagen, USA) followed by a DNase treatment using DNA-free^TM^ DNase kits (Ambion, USA). Complementary DNA (cDNA) synthesis and subsequent quantitative real-time polymerase chain reactions (qRT-PCRs) were carried out as described previously^60^. The primers were designed to target the total CpSRP43 mRNA (SRP43_F1 and SRP43_R1). The housekeeping gene 18 S rRNA was used as a loading control and was amplified by 18S_F2 and 18S_R2 primers (Table S1). The mean fold changes in gene expression compared to the wild type was calculated by 2^−ΔΔCT^ method^61^.

Protein samples were prepared as previously described^20^ and loaded into Any kD mini-protean TGX Stain-Free protein gels (Bio-Rad, USA). Separated proteins on the gels were transferred to polyvinylidene difluoride (PVDF) membranes (Bio-Rad, USA) using Trans-Blot Turbo kits (Bio-Rad, USA). The membranes were blocked with a 5% (*w*/*v*) skim milk solution dissolved in phosphate-buffered saline (PBS) containing 0.1% (*v*/*v*) Tween 20 (Sigma-Aldrich, Korea). The proteins were probed using the following antibody dilutions: anti-CpSRP43 (previously manufactured by our group^36^) at 1:1,000, anti-DYKDDDDK AS15-2871 at 1:1,000, LHCB1 AS01-004 at 1:4,000, LHCB2 AS01-003 at 1:10,000, LHCB3 AS01-002 at 1:2,000, LHCB4 AS06-117 at 1:5,000, PsbA AS05-084 at 1:10,000, PsaA AS06-72 at 1:2,000 or AtpB AS05-085 at 1:5000 (Agrisera, Sweden). The antibodies against LHCB1–3 were from *Arabidopsis thaliana* and the antibodies against CpSRP43, LHCB4, PsbA, PsaA and AtpB was from *Chlamydomonas reinhardtii*. Anti-mouse IgG-horseradish peroxidase (HRP) conjugated antibody AS11-1772 (Agrisera, Sweden) or anti-rabbit IgG-HRP conjugated antibody #7074 S (Cell Signaling Technology, USA) were then used as treatments at a dilution of 1:1,000. With an enhanced chemiluminescence substrate (Bio-Rad, USA), the signals were detected by the ChemiDoc system (Bio-Rad, USA). Band intensity was calculated based on densitometry using Image Lab program (Bio-Rad, USA).

### Analysis of photosynthetic parameters

Photoautotrophically grown cells in the mid-exponential growth phase were subjected to measurements of the photosynthetic parameters *in vivo* by means of Multi-Color-PAM (Heinz Walz, Germany). After 20 min for adaptation to the dark condition, the light response curves of the relative electron transport rate (rETR) and the yields of non-photochemical quenching (Y(NPQ)) were measured while increasing the actinic light intensities of 440 nm LEDs with a step width of 2 min.

### Analysis of cellular growth under different light intensities

Seed cultures of wild type *C*. *vulgaris*, the mutant strain E5 and the complemented strains were grown photoautotrophically in the BG-11 medium^50^ in 250 mL baffled flasks for 7 days, to adapt cells to different light conditions. Then each seed culture was inoculated to 400 mL column photobioreactor with a working volume of 200 mL which was illuminated with white LEDs at light intensities of 50 and 300 μmol photons m^−2^ s^−1^. CO_2_ (3% *v*/*v*) was constantly supplied at 0.25 VVM during the whole experiment. Cellular growth was monitored by measuring the optical density at 750 nm (*OD*_750_) and the cell density.

## Electronic supplementary material

Supplementary Information
