# Supplementary material for: Complementation of a mutation in CpSRP43 causing partial truncation of light-harvesting chlorophyll antenna in Chlorella vulgaris
Source: Sci Rep. 2017 Dec 20;7:17929. doi: 10.1038/s41598-017-18221-0 (PMC5738337; doi:10.1038/s41598-017-18221-0)
Supplement: Supplementary file 1 — Supplementary Information [file 41598_2017_18221_MOESM1_ESM.pdf]

Complementation of a mutation in *CpSRP43* causing the partial truncation of light-harvesting chlorophyll antenna in *Chlorella vulgaris*

Won-Sub Shin[1,2], Bongsoo Lee[1,2], Nam Kyu Kang[1,2], Young-Uk Kim[3], Won-Joong Jeong[3], Jong-Hee Kwon[4], Byeong-ryool Jeong[1,2*], Yong Keun Chang[1,2*]

[1]Advanced Biomass R&D Center (ABC), Korea Advanced Institute of Science and Technology (KAIST), Daejeon 34141, Republic of Korea

[2]Department of Chemical and Biomolecular Engineering, Korea Advanced Institute of Science and Technology (KAIST), Daejeon 34141, Republic of Korea

[3]Plant Systems Engineering Research Center, Korea Research Institute of Bioscience and Biotechnology (KRIBB), 125 Gwahak-ro, Yuseong-gu, Daejeon 34141, Republic of Korea

[4]Department of Food Science and Technology, Institute of Agriculture and Life Science, Gyeongsang National University, Jinju 660-701, Republic of Korea

*Correspondence and requests for materials should be addressed to B.-r.J. (email: bjeong@kaist.ac.kr) or Y.K.C. (email: changyk@kaist.ac.kr)

Table S1. List of primers used in this study

Primer name	Primer sequence (5’ to 3’)
SRP43_Gibson_fwd	CGCTTCTGTCACCTGCAACCATGGCGTCGGCAACCT
SRP43_Gibson_rev	TCACTTGTCGTCGTCGTCCTTGTAGTCCGATGGCGAGCCTTGCA
pUC19_fwd	GACTACAAGGACGACGACGACAAGTGAGAGCTCGAATTTCCCCGATCGTT
pUC19_rev	GTGCAGGTTGCCGACGCCATGGTTGCAGGTGACAGAAGCG
Aph_Gibson_fwd	TAGATCGGGAATTCACTGGCTTCTTGCGCTATGACACTTC
Aph_Gibson_rev	CACGACGTTGTAAAACGACGGAAAGAGGCCAAAATCAACG
Aph-pUC_back_fwd	CGTTGATTTTGGCCTCTTTCCGTCGTTTTACAACGTCGTG
Aph-pUC_back_rev	GAAGTGTCATAGCGCAAGAAGCCAGTGAATTCCCGATCTA
S4_F	CGACTGCTGGTTACTCCACT
S4_R	AGTCTCCTCGTCTCCCTTCTT
SRP43_F1	CAGAGGTGGCGGATCTGAAG
SRP43_R1	GTCTCCCTTCTTCACGGCTC
18S_F1	ACTTCACGAATCGCATGGCCT
18S_R1	GACTTGCCCTCCAATTGATCC
18S_F2	GAGTATGGTCGCAAGGCTGA
18S_R2	ACCCACCAACTAAGAACGGC

Table S2. Assessment of genomic sequence qualities and identification of polymorphic single nucleotide polymorphism (SNP) loci

# of CDS gene	# of CDS gene comparable to reference	# of CDS gene including SNP	# of SNP detected in CDS gene	# of polymorphic SNP loci between WT and E5	# of polymorphic SNP loci in CDS
9,427	9,016	212	533	63	20

Table S3. Annotations of SNPs of WT and E5 in the coding sequence

				**BLAST Search**			
**WT amino acid change**	**Ref vs WT**	**E5 amino acid change**	**Ref vs E5**	**E-value**	**Identity (%)**	**Accession**	**Gene description (Organism name; Gene ID)**
GTG(V)->GTG(V)	SYN	GTG(V)->GAG(E)	NONSYN	9.00E-04	56.82	AGC59877.1	chloroplast SRP43 (*Chlamydomonas reinhardtii*)
TGG(W)->TGG(W)	SYN	TGG(W)->TTG(L)	NONSYN	2.00E-05	75	XP_008457400.1	PREDICTED: LOW QUALITY PROTEIN: helicase SKI2W (*Cucumis melo*)
AGG(R)->AGG(R)	SYN	AGG(R)->AAG(K)	NONSYN	0.7	37.93	XP_010062894.1	PREDICTED: uncharacterized protein LOC104450151 (*Eucalyptus grandis*)
AGC(S)->AGC(S)	SYN	AGC(S)->AAC(N)	NONSYN	3.2	43.18	KFY86180.1	hypothetical protein (*Pseudogymnoascus pannorum* VKM F-4518 (FW-2643); V500_07812)
AGA(R)->AGA(R)	SYN	AGA(R)->AAA(K)	NONSYN				
ACT(T)->ACT(T)	SYN	ACT(T)->ATT(I)	NONSYN	5.2	31.34	XP_007303555.1	hypothetical protein (*Stereum hirsutum* FP-91666 SS1; STEHIDRAFT_110529)
CGG(R)->CGG(R)	SYN	CGG(R)->TGG(W)	NONSYN				
GTG(V)->GTG(V)	SYN	GTG(V)->GTA(V)	SYN				
CTG(L)->CTG(L)/CTA(L)	SYN	CTG(L)->CTG(L)	SYN	4	33.33	KFA80735.1	hypothetical protein (*Stachybotrys chartarum* IBT 40288; S40288_11671)
CTG(L)->CTG(L)	SYN	CTG(L)->CTA(L)	SYN	1.00E-10	81.08	XP_013906390.1	Ubiquitin and WLM domain-containing protein (*Monoraphidium neglectum*)
CTC(L)->CTC(L)	SYN	CTC(L)->CTT(L)	SYN				
CGC(R)->CGT(R)	SYN	CGC(R)->CGC(R)	SYN	3.00E-04	58.33	XP_005849359.1	expressed protein (*Chlorella variabilis*)
AAG(K)->AAA(K)	SYN	AAG(K)->AAG(K)	SYN				
AAC(N)->AAC(N)	SYN	AAC(N)->AAT(N)	SYN	1.4	26.05	KIO21807.1	hypothetical protein (*Tulasnella calospora* MUT 4182; M407DRAFT_28620)
TCG(S)->TCG(S)/TTG(L)	NONSYN	TCG(S)->TCG(S)	SYN	2.00E-62	62.43	XP_013898255.1	B12-dependent methionine synthase (*Monoraphidium neglectum*)
CGC(R)->TGC(C)	NONSYN	CGC(R)->CGC(R)	SYN	3.00E-26	35.82	BAB18104.1	cyclic nucleotide dependent protein kinase (*Chlamydomonas reinhardtii*)
CGC(R)->CAC(H)	NONSYN	CGC(R)->CGC(R)	SYN	9.00E-19	67.24	XP_011401820.1	Zinc finger protein (*Auxenochlorella protothecoides*)
CAG(Q)->TAG(*)	NONSYN	CAG(Q)->CAG(Q)	SYN	0.57	45.45	XP_002339873.1	Pfs, NACHT and WD domain protein (*Talaromyces stipitatus* ATCC 10500)
TCT(S)->TTT(F)	NONSYN	TCT(S)->TCT(S)	SYN	3.00E-08	65.96	XP_011399816.1	Desiccation-related protein PCC13-62 (*Auxenochlorella protothecoides*)
CTA(L)->CAA(Q)/CTA(L)	NONSYN	CTA(L)->CAA(Q)	NONSYN	3.00E-77	63.13	XP_013898255.1	B12-dependent methionine synthase (*Monoraphidium neglectum*)

Abbreviations: SYN: Synonymous; NONSYN: Nonsynonymous

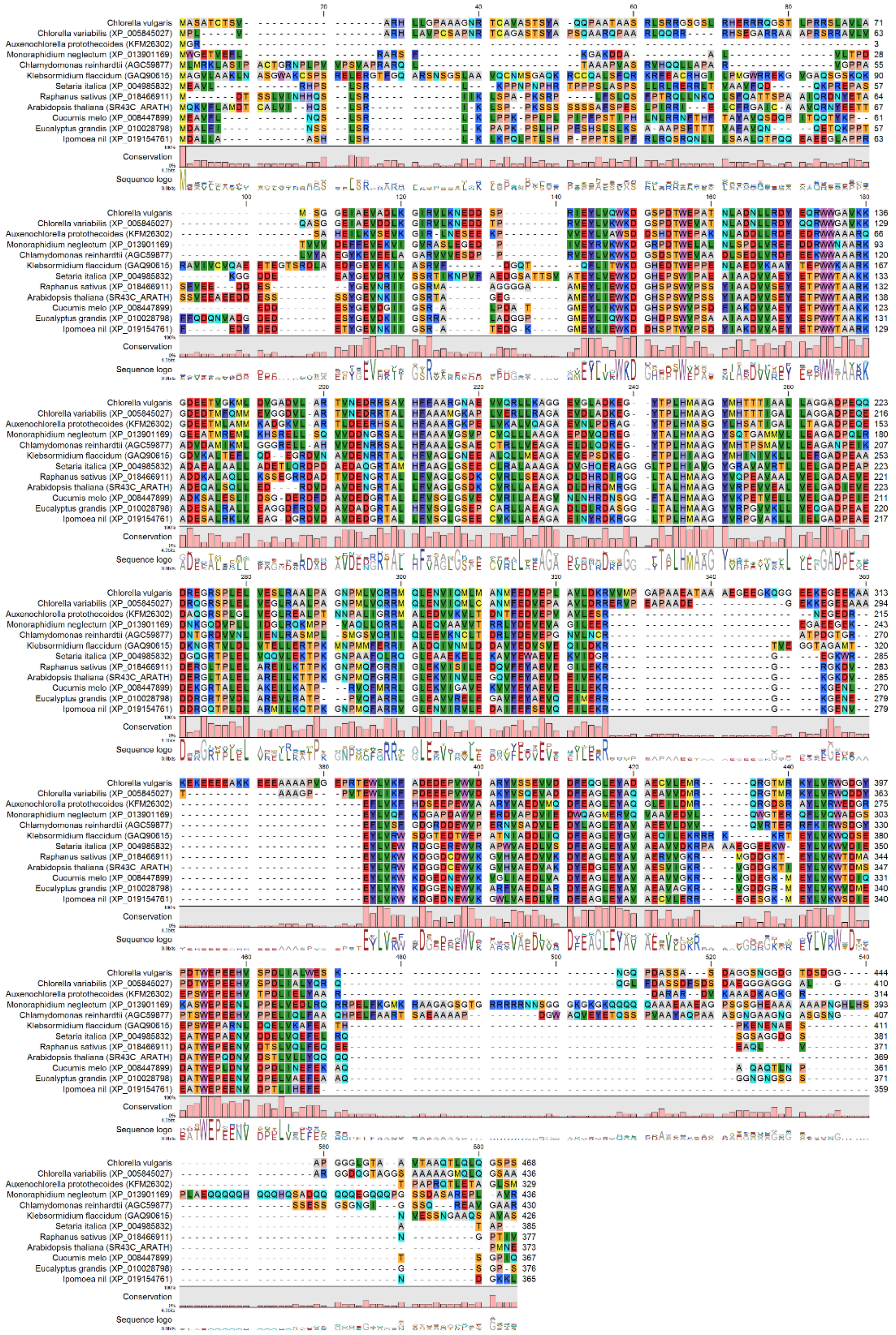

Fig. S1. Alignments of CpSRP43 homologs from plants and microalgae. The protein sequences were downloaded from NCBI (https://www.ncbi.nlm.nih.gov/). Background colors of amino acid and conserved sequence logo were determined according to Rasmol color scheme (http://www.openrasmol.org/doc/rasmol.html).

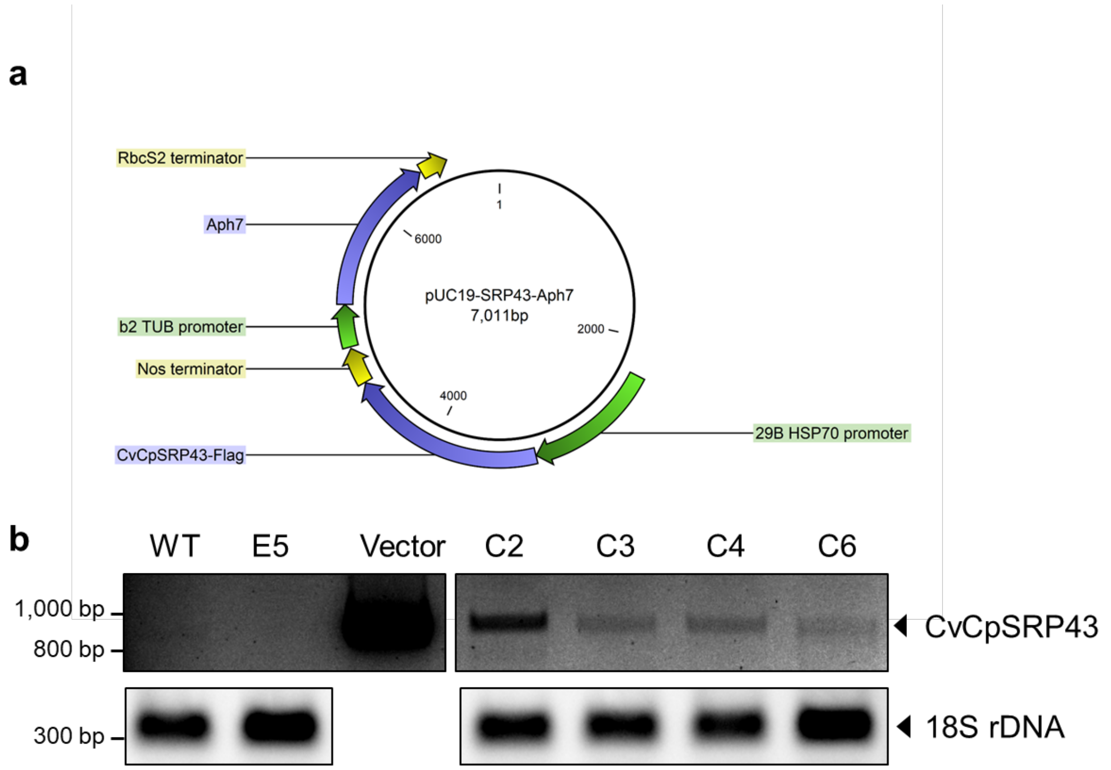

Fig. S2. Vector map and confirmation of vector transformation. (a) Vector used for complementation test of *C. vulgaris* E5, including coding sequence of the *CpSRP43* gene of *C. vulgaris* (CvCpSRP43). (b) Confirmation of vector transformation into the complemented strains (C2, C3, C4 and C6) by PCR. The amplification target size was 938 bp (upper) which includes both 29B HSP70 promoter and CvCpSRP43-Flag regions. 18S rDNA bands were detected as a control, and the amplification target size was 303 bp (lower). Images were cropped for better comparison.

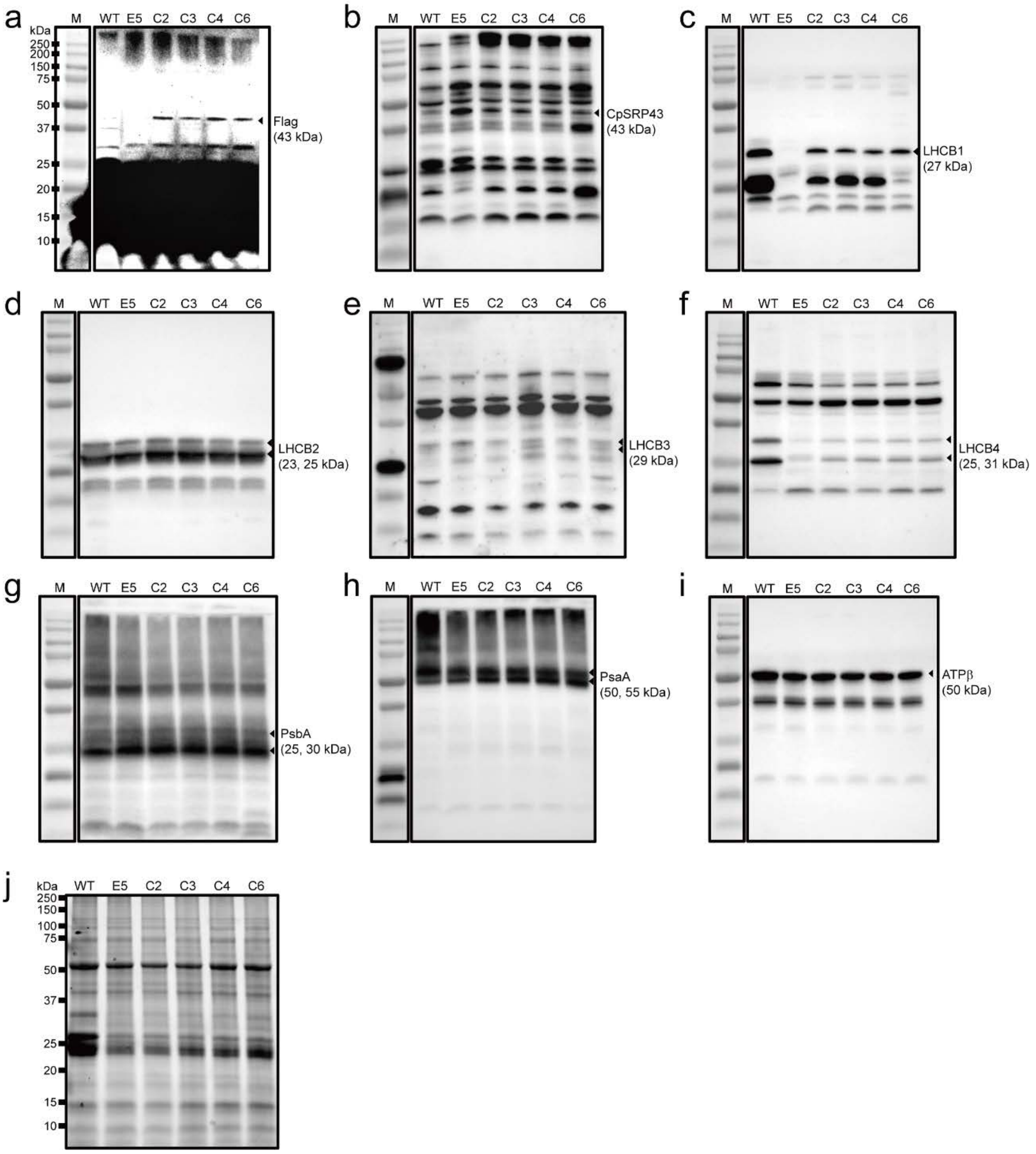

Fig. S3. Full-length gel and western blot images. Proteins were detected with (a) anti-Flag tag, (b) anti-CpSRP43, (c) anti-LHCB1, (d) anti-LHCB2, (e) anti-LHCB3, (f) anti-LHCB4, (g) anti-PSBA, (h), anti-PSAA and (i) anti-AtpB. Gel image of whole proteins before western blot was pictured by the ChemiDoc system (Bio-Rad, USA), which showed bands near 25kDa that might represent antenna proteins (j). Arrowheads indicate expected bands with predicted sizes. In several blots with multiple bands, slightly lower bands near expected bands were considered as degraded products and also marked as target proteins. Prestained markers (M) were imaged separately and combined with the blot images.

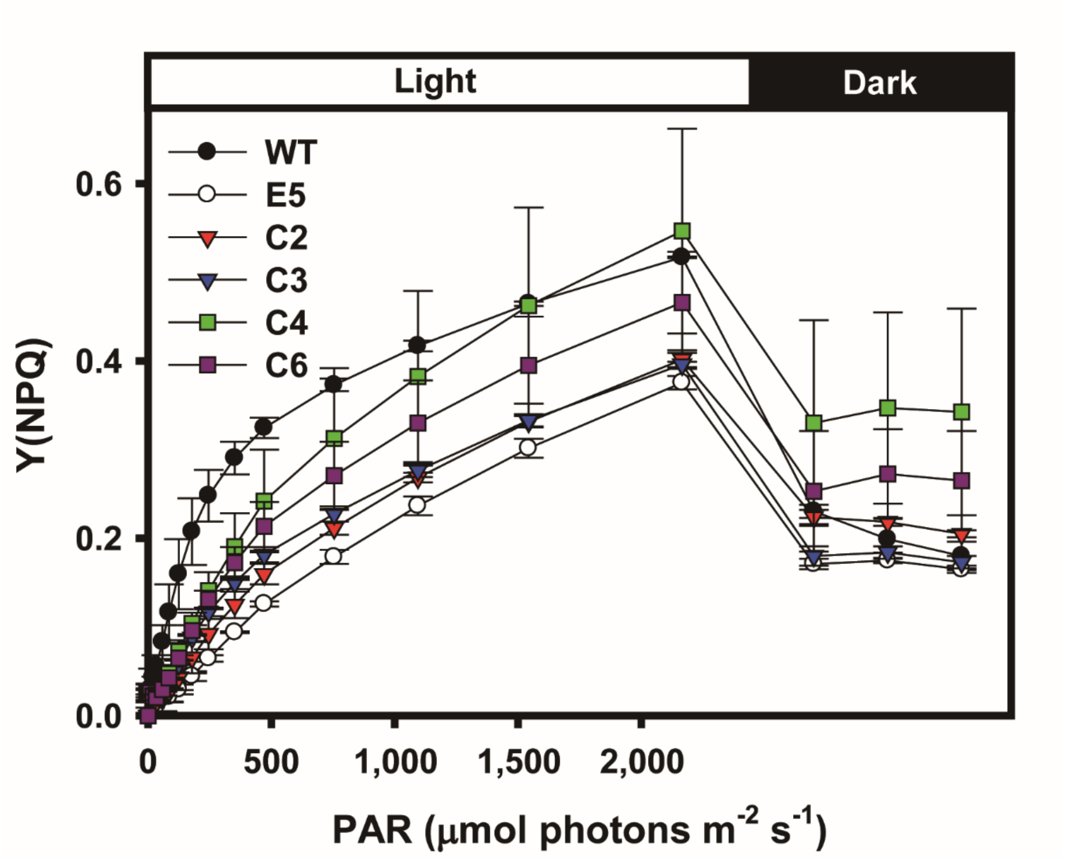

Fig. S4. Effect of complementation on non-photochemical quenching. Light-dependent curves of quantum yields of the non-photochemical quenching (Y(NPQ)) of wild type, E5 and complemented strains (C2, C3, C4 and C6). The white bar at the top of the figure indicates the illumination period with an increase in the actinic light, and the dark bar indicates the dark recovery after illumination. The step width of each data point was 2min. The data represent the average of samples and the error bars indicate the standard error (*n*=2).
